# Facile synthesis of Bi_2_O_3_/BiOX mixed-phase for electrochemical detection of paracetamol

**DOI:** 10.1039/d6ra01611a

**Published:** 2026-03-31

**Authors:** Raamisa Anjum, Md. Hasanuzzaman, Muhammad Shahriar Bashar, Juliya Khanam, Umme Sarmeen Akhtar, A. M. Sarwaruddin Chowdhury, Samina Ahmed, Sumaya Farhana Kabir, Md. Sahadat Hossain

**Affiliations:** a Department of Applied Chemistry and Chemical Engineering, University of Dhaka Dhaka-1000 Bangladesh sumaya.kabir@du.ac.bd; b Institute of Glass & Ceramic Research and Testing, Bangladesh Council of Scientific and Industrial Research (BCSIR) Dhaka 1205 Bangladesh shanta_samina@yahoo.com saz8455@gmail.com; c Institute of Energy Research & Development, Bangladesh Council of Scientific and Industrial Research (BCSIR) Dhaka Bangladesh

## Abstract

In recent times, paracetamol (*N*-acetyl-*p*-aminophenol) detection has become crucial due to its widespread clinical use combined with the public health issue of hepatotoxicity from overdose. In this research, mixed-phase Bi_2_O_3_/BiOX (X = Cl, Br and I) was synthesized at room temperature in a single-step solid–state reaction and for the first time used in paracetamol detection. Field emission scanning electron microscopy (FESEM), X-ray diffractometry (XRD), Fourier transformed infra-red (FTIR), Thermogravimetric analysis (TGA), Differential scanning calorimetry (DSC) and UV-visible Spectrophotometry were performed to determine the existence of a double phase in the compounds and to specify the characteristics. Different electrochemical methods were performed using a glassy carbon electrode modified with Bi_2_O_3_/BiOX to sense paracetamol. The highest surface area of 3.496 cm^2^ was found for the Bi_2_O_3_/BiOCl electrode which showed an electron transfer coefficient (*α*) of 0.4 and electron transfer rate constant of 0.12 cm s^−1^ for the process. As Bi_2_O_3_/BiOCl showed a superior response to the other samples, the limit of detection (LOD) = 2.24 ppm, as well as the limit of quantification (LOQ) = 7.47 ppm was determined in a linear concentration range of 1–50 ppm by cyclic voltammetry (CV). Paracetamol detection was also inspected by pulse voltammetry at a linear concentration range of 0.5–5 ppm. The sensitivity of the electrode was found to be 0.9776 µA ppm^−1^, coupled with excellent reproducibility.

## Abstract 2

Bi_2_O_3_/BiOX mixed phase semiconductor for electrochemical sensing of paracetamol.

## Graphical abstract

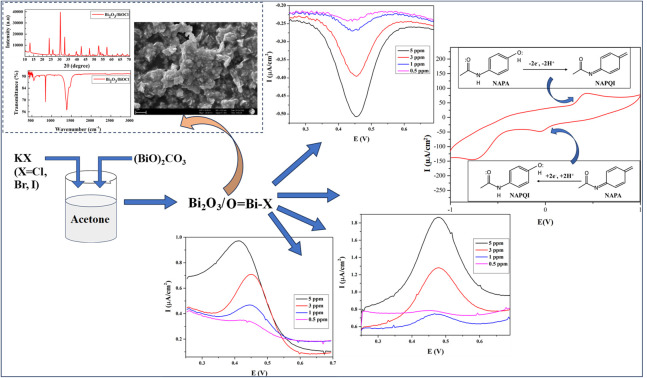

## 1. Introduction

Paracetamol (acetaminophen) is one of the most available and inexpensive over-the-counter (OTC) analgesics and antipyretics. It has been prescribed as a pain reliever for mild to moderate headaches, muscle aches, toothaches, menstrual cramps, backaches, arthritis, *etc.* and as a cold and flu medication to reduce fever. Due to high demand, the volume of paracetamol production surpassed 100 000 MT per year a long time ago.^1^ The vast use of paracetamol also leads to its introduction into the environment by incomplete human metabolism, pharmaceutical discharge, hospital and municipal wastes and unauthorised disposal.^2^ As paracetamol is highly soluble in water and slightly biodegradable in nature, it has been found to accumulate in the environment at concentrations as high as 65 µg L^−1^.^3^ Due to its frequent presence in groundwater, surface water and wastewater, paracetamol has been classified as an aquatic organic micropollutant that can give rise to significant risks to aquatic organisms and human beings.^4,5^ Furthermore, it can be transmitted through the food chain as paracetamol can accumulate in soil and plants, especially leafy vegetables.^6^ Its easy access without prescription and environmental persistence can result in overdosing, which can cause acute and chronic damage like hepatoxicity, renal failure, *etc.*^7–10^

For these reasons, precise detection of paracetamol is a critical requirement to avoid any risk from overdosing. To determine the level of paracetamol, several different methods like UV-Vis spectrophotometry, chromatographic methods such as liquid chromatography (LC), thin-layer chromatography (TLC), gas chromatography (GC), high-performance liquid chromatography (HPLC), voltammetric methods, capillary electrophoresis, electrochemical methods, *etc.* are in use.^11–14^ Among these, electrochemical methods have drawn significant attention due to their simplicity of operation, high sensitivity, lower cost and fast responsiveness.^15,16^ Electrochemical sensors have become increasingly important for the sensitive detection of different compounds. Recent studies have explored various electrochemical approaches for sensing purpose, demonstrating enhanced analytical performance in complex matrices.^17–19^ Graphite, glassy carbon, screen printed, carbon paste, *etc.* are various types of electrodes that are being used for this purpose and have been modified with metals, metal oxides, nanocarbons, composites, polymers, dyes and so on.^20–22^ Nanomaterials, especially those made from noble metals like gold, platinum, and palladium, show extraordinary electrocatalytic properties, but their application is limited by high cost.^23^ However, metal oxides have been used as efficient alternatives, as these have shown enhanced activity due to structural defects.^24^ Bi_2_O_3_, which is a well-known transition metal oxide, has gained notable interest because of its energy band gap, large surface area, and high electrochemical stability^25^ and has been used in the detection of paracetamol, ascorbic acid and heavy metals.^26^ Another class of Bi is bismuth oxyhalides (BiOX, X= Cl, Br, I), which have been extensively studied as photocatalysts due to their unique optical, electronic, and structural properties.^27^ Recently, these have been studied for electrochemical analysis and have shown success in the detection of molecules,^28^ water splitting reaction,^29^ CO_2_ reduction reaction (CO_2_RR)^30^ and oxygen evolution reaction (OER).^31^

Although Bi_2_O_3_ and BiOX individually show excellent electrocatalytic activities, there is no report of combined application associating these materials in paracetamol sensing. In this study, an attempt was made to synthesize mixed-phase Bi_2_O_3_/BiOX composites by a simple one-step method without implementing high temperature and excess reagents. The effect produced by the presence of both phase and different halide ions on electrochemical properties was investigated by various voltametric methods to monitor the detection process and levels of paracetamol.

## 2. Methodology

Bismuth subcarbonate was reacted with potassium halide salts (KCl, KBr and KI) to prepare the mixed phase Bismuth oxide/bismuth halide (Bi_2_O_3_/BiOX) samples. (BiO)_2_CO_3_ (M.W. 509.97, purity 80%) was purchased from Sisco Research Laboratories Pvt. Ltd, India. All the KX salts were bought from Merck, Germany, with 99.5% purity. To prepare the biphasic samples, (BiO)_2_CO_3_ was reacted with KX at a molar ratio of 1 : 2 in acetone and stirred for 4 hours in a covered beaker. After that, the cover was removed, and further stirring was continued for 1 hour to evaporate acetone. All these steps were conducted inside a fume hood. The residual mass was washed with deionized water to remove K_2_CO_3_. After washing, drying was done at 110 °C for 5 hours in an oven.

### 2.1. Characterization

Rigaku SmartLab SE X-ray diffraction machine (Japan) was used for lattice structure and phase analysis utilizing Cu K_α_ (*λ* = 1.5406 Å) radiation at 50 mA – 40 kV, over the 2*θ* range of 10–70°. The obtained data were investigated by SmartLab Studio II software. Fourier transform infrared (FTIR) spectroscopy for functional group assessment was accomplished by IR Prestidge 21 (Shimadzu, Japan), and the surface morphology was studied using a scanning electron microscope (SEM, EVO18, Zeiss) that was operated at 25 kV. The XPS analysis was performed using the Thermo Scientific K-Alpha instrument. Thermogravimetric analysis (TGA) and Differential Scanning Calorimetry (DSC) were investigated by the NETZSCH STA 449 F5 instrument. PerkinElmer Lambda 1050+ UV-visible Spectrophotometer was employed to understand the Optical bandgap energy. An impedance analyzer from Wayne Kerr Electronics, model: 65120B, UK was used to analyze the dielectric properties of the sample within a specific frequency range of 20 Hz to 20 MHz.

### 2.2. Electrochemical study

An electrochemical instrument of the CS300 model from the Corrtest manufacturer (China) was used to carry out all the electrochemical analyses documented in this paper. The three-electrode system composed of a reference electrode (Ag/AgCl), working electrode (glassy carbon-GC) and counter electrode (platinum wire) was chosen to conduct the investigations. The modification of the GC electrode was performed using a 1 : 1 (w/v) mixture of synthesized Bi_2_O_3_/BiOX in acetone. 10 µl solution was dropped on the bare GC electrode surface, and sufficient time was provided to completely evaporate the acetone. 0.1 M NaCl was implemented as a supporting electrolyte over the entire electrochemical experimentation.

## 3. Results and discussions

**Fig. 1:**
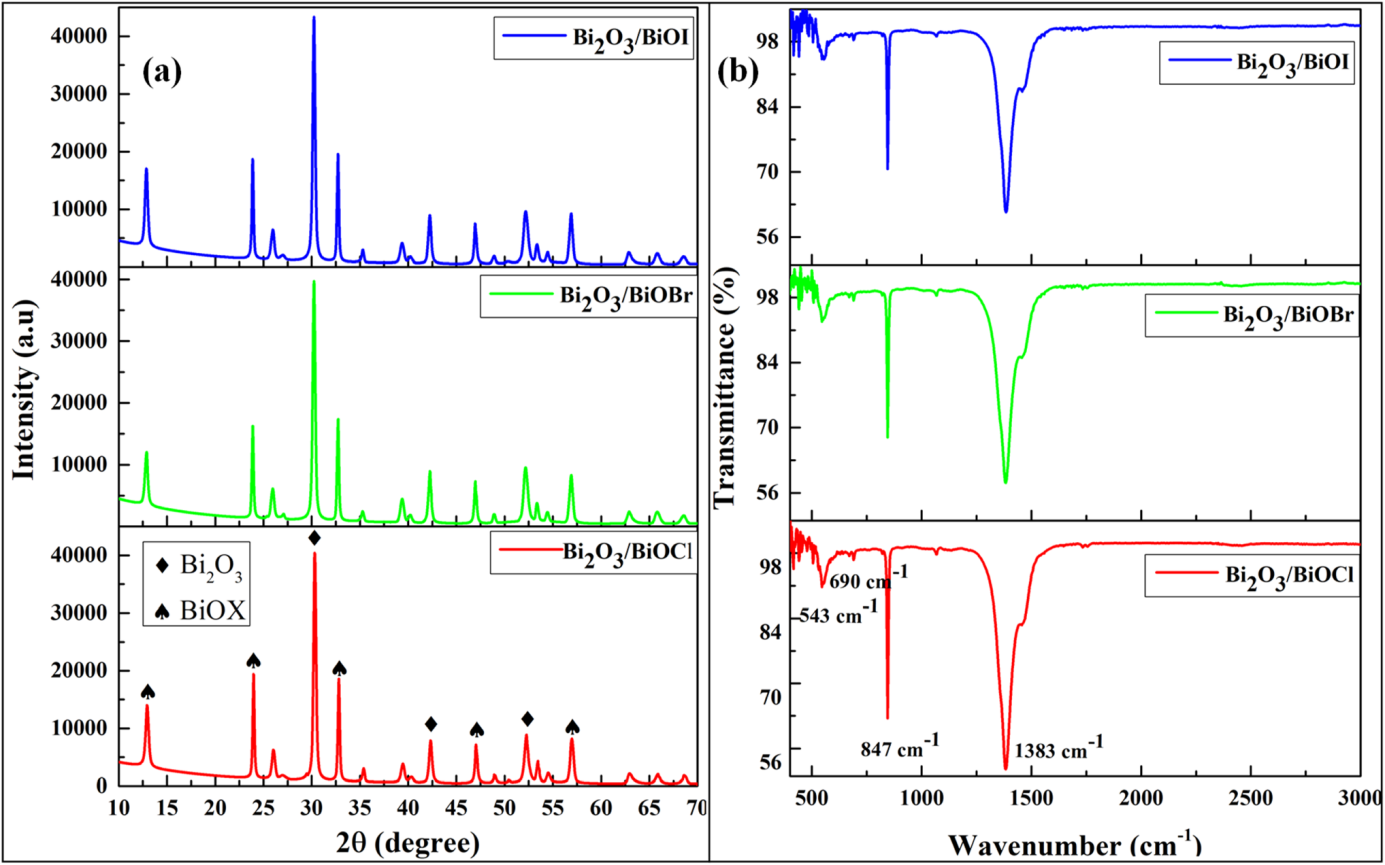
Characterization of the synthesized samples by (a) XRD and (b) FTIR.

Fig. 1(a) displays the X-ray diffraction (XRD) patterns of the Bi_2_O_3_/BiOX samples with distinct sharp peaks. All three samples showed peaks of Bi_2_O_3_ at 2*θ* angles of 30.2, 42.3 and 52.2°, corresponding to the (2 2 2), (3 3 2), and (4 3 3) planes, respectively.^32^ The samples were found to have similar patterns, but the peaks corresponding to specific diffraction planes gradually shifted to lower 2*θ* values as chloride was replaced by bromide and iodide in the samples. For example – 2*θ* values for (0 0 1), (0 0 2), (1 1 0), (2 0 0) and (2 1 2) planes were found respectively, at 12.93, 23.98, 32.80, 47.02 and 56.93° for Bi_2_O_3_/BiOCl which slightly shifted to 12.88, 23.89, 32.73, 46.96 and 56.89° for Bi_2_O_3_/BiOBr and then to 12.85, 23.85, 32.72, 46.93 and 56.88° for Bi_2_O_3_/BiOI.^33,34^ This can be explained by the ionic radius, as the bromide ion (0.196 nm) is larger in size than the chloride ion (0.181 nm), and again the iodide ion (0.219 nm) is the largest among them.^35^ Due to this factor, an increase in interplanar distance resulted in a shift of diffraction angles.

The FTIR analysis is shown in Fig. 1(b) is used to examine the presence of functional groups in the prepared samples. The presence of a peak at 1383 cm^−1^ indicated the presence of Bi-X bonds in the samples.^24^ The other peaks at 543 and 847 cm^−1^ were caused by the asymmetric stretching vibration of Bi–O bonds, and the peak present at 690 cm^−1^ referred to the Bi–O stretching.^36^

**Fig. 2:**
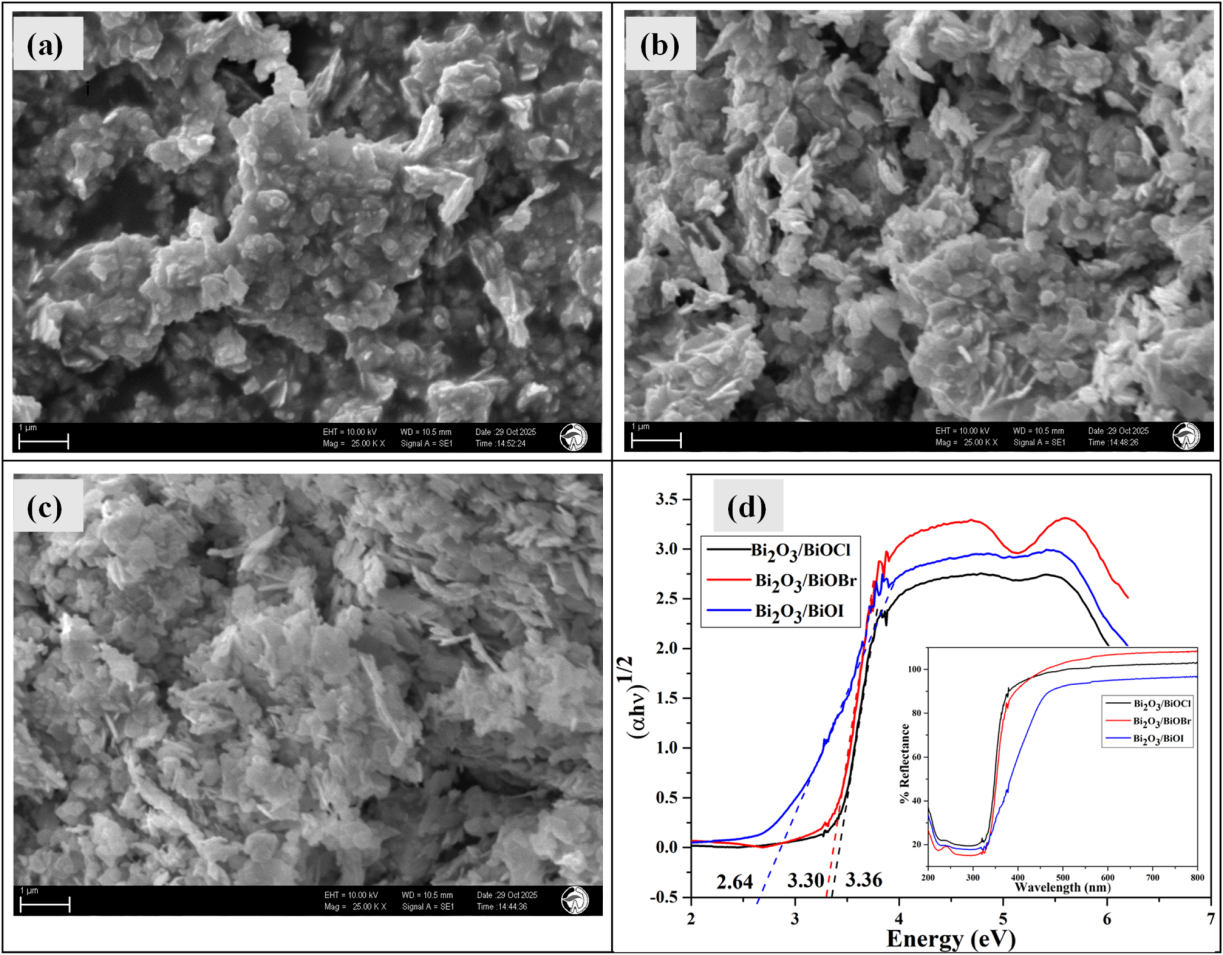
SEM image of (a) Bi_2_O_3_/BiOCl, (b) Bi_2_O_3_/BiOBr, (c) Bi_2_O_3_/BiOI; and (d) UV-Vis DRS of the samples and corresponding Kubelka–Munk plots.

The morphological analysis of the surface was done by SEM, which is shown in Fig. 2. The surface morphology of Bi_2_O_3_/BiOCl (Fig. 2(a)) revealed an aggregated porous structure, mostly made of plate-like flakes. With the presence of bromine, the Bi_2_O_3_/BiOBr flakes were found to become thinner and grow in the outward direction (Fig. 2(b)). The aggregation was still present, but the pores were visibly reduced. The surface of Bi_2_O_3_/BiOI (Fig. 2(c)) was fully comprised of very thin, long, flake-like aggregated structures and the pores were minimized to a significant extent. To further examine the elemental composition, EDS analysis (Fig. S1(a)–(c)) was performed. The spectra confirmed the presence of Bi, O and corresponding halogen elements, supporting the successful formation of Bi_2_O_3_/BiOX. The halogen content increased progressively from Cl to Br to I. This may have occurred due to the surface limited distribution of Cl, which resulted in a lower detected percentage by EDS, whereas heavier halogens (Br and I) were more embedded in the material. This may also have caused the reduction in porosity of the structure, which aligns with the SEM analysis. Moreover, partial loss of chloride during washing or processing can contribute to the lower percentage of Cl.

Fig. 2(d) shows the UV-Vis diffuse reflectance spectrum and the corresponding Kubelka–Munk function *vs.* photon energy plots in the 200–800 nm wavelength range. It is visible that all the samples strongly absorbed in the UV region with an edge around 315–330 nm and went into an optically inert state in the visible region. The band gap was calculated using the Kubelka–Munk equation (eqn (1)):^37^

1 (*αhν*) = *A*(*hν* − *E*_g_)^*n*/2^

Here *α* denotes the absorption coefficient, *E*_g_ is the band gap energy, *ν* represents light frequency, *A* defines a constant, and *h* is Planck's constant. As it is known that BiOX show an indirect band gap, the value of *n* was taken to be 4 and (*αhν*)^1/2^*vs.* (*hν*) was plotted.^38^ The band gap energy was estimated for each sample and found to be 3.35, 3.31 and 2.65 eV for Bi_2_O_3_/BiOCl, Bi_2_O_3_/BiOBr and Bi_2_O_3_/BiOI, respectively. The obtained band gaps were higher than the band gaps of individual bismuth oxyhalides,^39^ which may have been caused by the mixed phase structure.

**Fig. 3:**
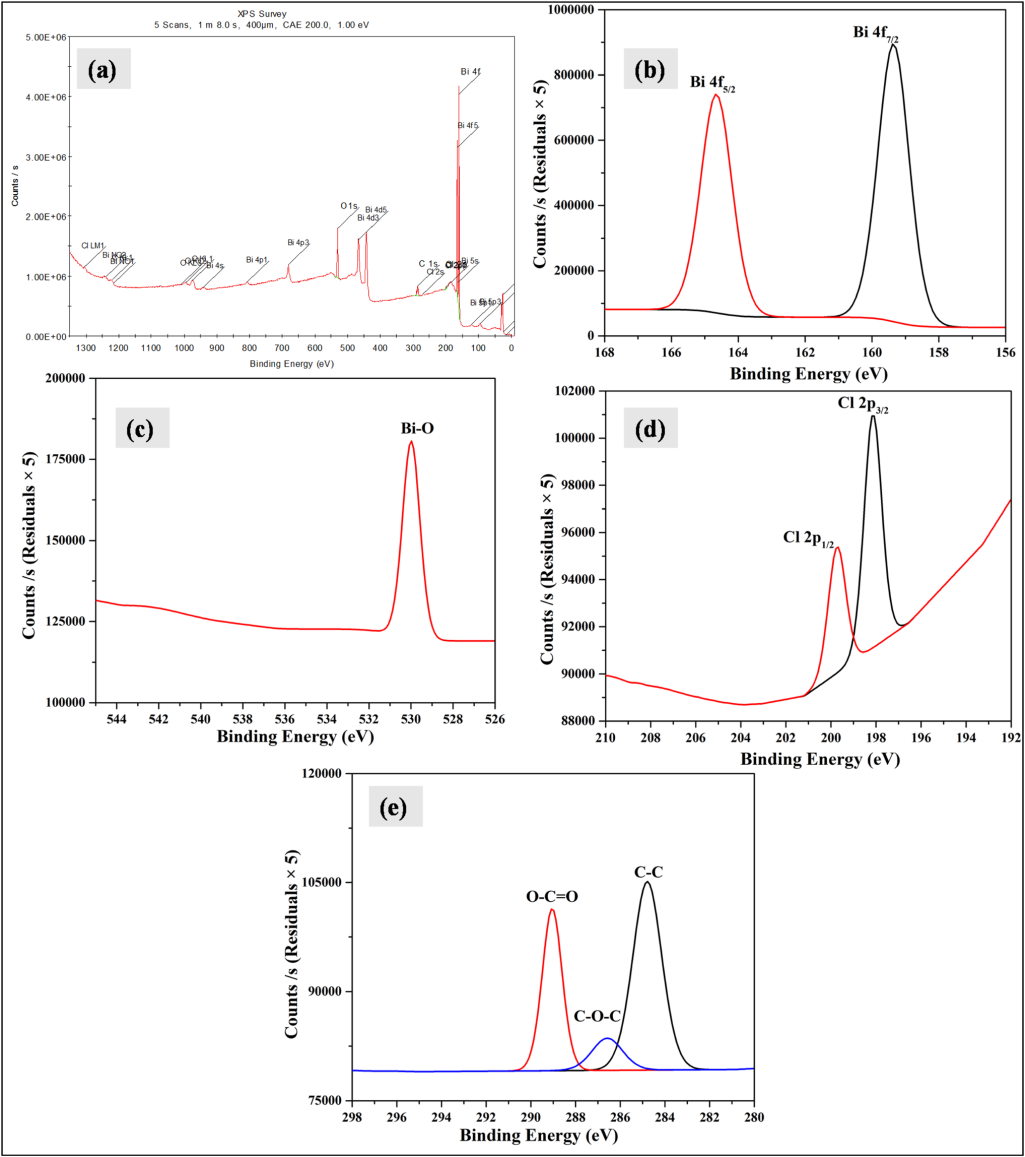
XPS analysis of Bi_2_O_3_/BiOCl sample; (a) XPS survey, and narrow scan for (b) Bi, (c) O, (d) Cl and (e) C.

XPS analysis was employed to investigate the chemical state of Bi_2_O_3_/BiOCl. The survey spectrum (Fig. 3(a)) confirms the presence of Bi, O, and Cl, along with a minor C signal, indicating the successful formation of the Bi_2_O_3_/BiOCl phase. The narrow spectrum of Bi 4f (Fig. 3(b)) exhibited two characteristic peaks corresponding to Bi 4f_7/2_ and Bi 4f_5/2_ at binding energies around 159.38 eV and 164.58 eV, respectively and the peak-to-peak separation was found to be 5.2 eV which is consistent with Bi^3+^ oxidation states and suggests the presence of bismuth oxide and bismuth oxyhalide phases without metallic bismuth.^40^ The FWHM were calculated to be 1.12 and 1.29 eV for Bi 4f_7/2_ and Bi 4f_5/2_ peaks, respectively. The O 1s spectrum (Fig. 3(c)) showed multiple binding energies, among which the M-oxide (Bi–O bonds) showed intense peak at 529.98 eV. The FWHM for this peak was 1.02 eV. Fig. 3(d) shows the peaks corresponding to Cl 2p_3/2_ and Cl 2p_1/2_ appearing at the characteristic binding energies of 198.18 and 199.68 eV, which confirm the presence of Cl.^41^ The FWHM were 0.89 and 0.92 eV for Cl 2p_3/2_ and Cl 2p_1/2_. The peak-to-peak separation was found to be 1.5 eV, which matches well with the literature.^42^ Carbon that had been used for the calibration of the instrument also showed C 1s peaks (Fig. 3(e)) corresponding to C–C, C–O–C and O–C=O states at binding energies around 284.78, 286.58 and 289.08 eV, respectively. From the XPS study, the Cl composition in the Bi_2_O_3_/BiOCl sample was calculated to be 7.23%, which varies significantly from the EDS analysis. This variation confirms the distribution of Cl mostly on the surface rather than in the bulk, as XPS provides information about surface chemistry, where EDS focuses on bulk analysis at several micrometer depth in samples.^43,44^

**Fig. 4:**
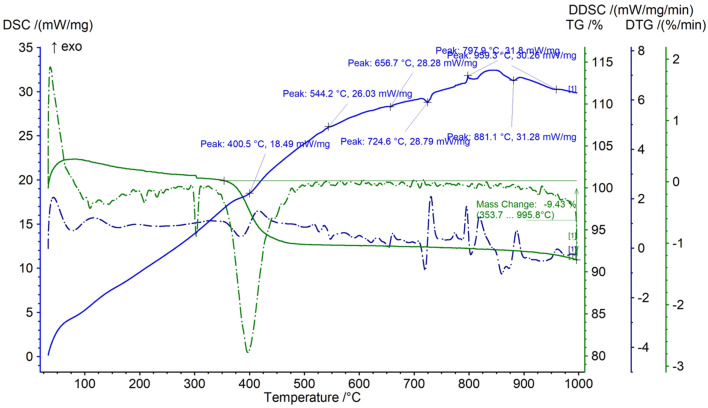
TGA and DSC plots of Bi_2_O_3_/BiOCl.

It was visible from the TGA analysis (Fig. 4) that the sample showed a sudden weight loss in the 350–450 °C range, with a very little mass loss till 1000 °C, resulting in a total loss of 10% mass. The DSC plot also showed a small but defined endothermic peak at a similar temperature range (350–450 °C). Previous research shows that BiOCl starts to convert into Bi_2_O_3_ around 400 °C by releasing Cl_2_.^45^ In addition to that, the presence of some unreacted (BiO)_2_CO_3_ may have caused CO_2_ evolution over the temperature span, leaving behind Bi_2_O_3_ solid.^46^ After this extent, there was no significant mass loss or heat loss, which resembles the thermally stable nature of the synthesized material.^47^

**Fig. 5:**
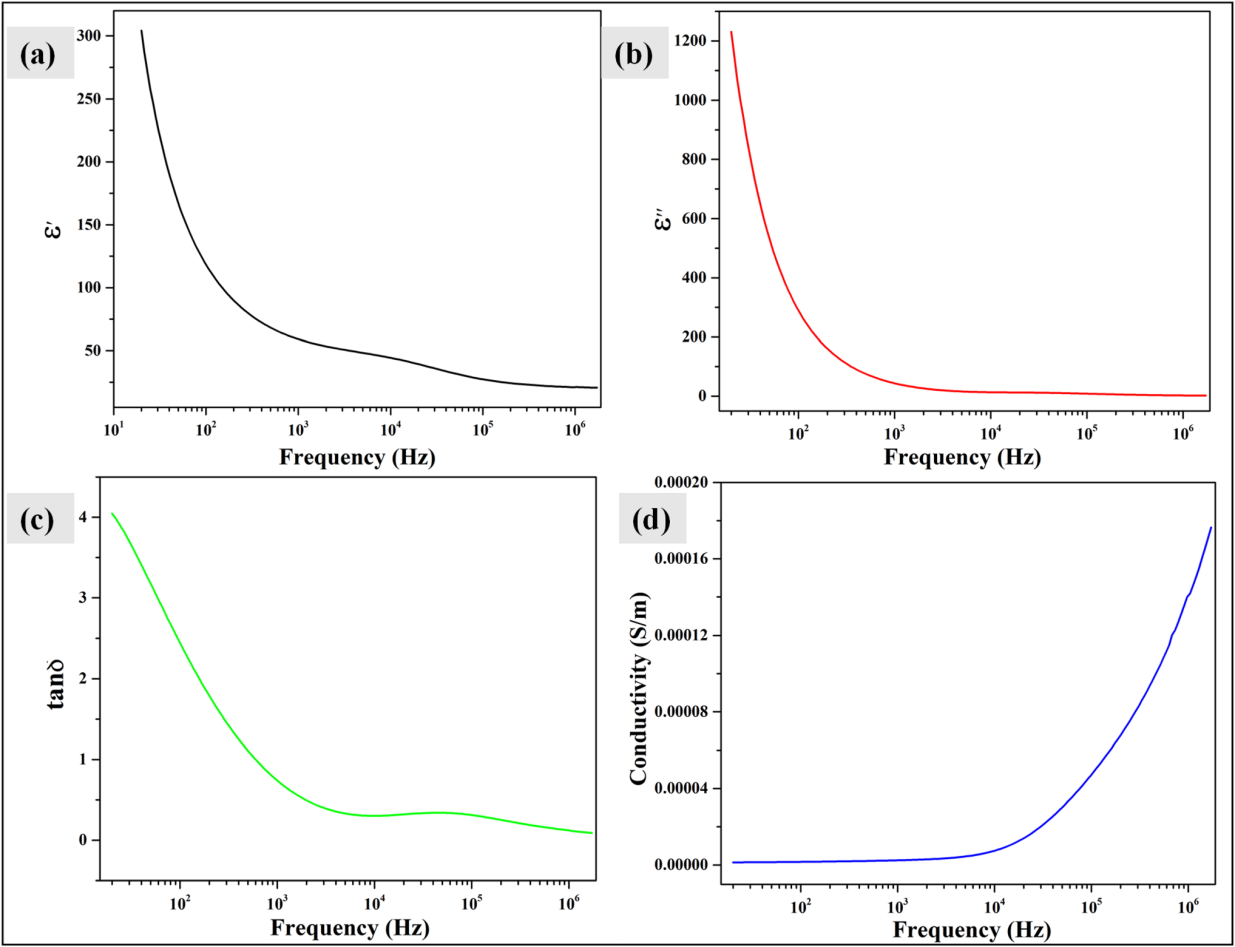
Dielectric analysis of Bi_2_O_3_/BiOCl sample; (a) real part, *ε*′ and (b) imaginary part, *ε*″ of the dielectric constant; (c) dielectric loss and (d) AC conductivity.

For Bi_2_O_3_/BiOCl sample, its dielectric properties were also assessed. Real (*ε*′) and imaginary (*ε*″) parts of the dielectric constant visualized in Fig. 5(a) and (b) were calculated using the following mathematical expressions (eqn (2) and (3)), where *ε*_0_ is the dielectric constant in vacuum (8.854 × 10^−12^ F m^−1^), A is the sample area, C stands for capacitance, *d* defines the sample thickness, and *D* denotes the dissipation factor:^48^2
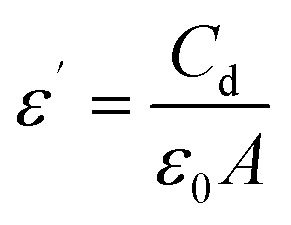

3 *ε*″ = *ε*′*D*

The dielectric constant showed frequency dependent behavior at room temperature and decreased with increasing frequency. High value of the dielectric constant at lower frequency region occurred due to space charge polarization as charge carriers are able to move and store according to the alternating electric field.^49^ A similar pattern was observed in the plot of dielectric loss (tan *δ*) *vs.* frequency graph (Fig. 5(c)). This happened because in the low frequency region, the grain boundaries stay more active compared to the grains. In this frequency range, charge carriers face more resistance due to grain boundaries, thus requiring higher energy for hopping and causing more energy loss than in the high frequency region.^50^Fig. 5(d) shows the frequency dependent AC conductivity (*σ*_AC_) of the material which was determined by eqn (4).^51^

4 *σ*_AC_ = 2π*fε*_0_*ε*′ tan *δ*

The AC conductivity of Bi_2_O_3_/BiOCl gradually increased with rising frequency which can be explained by the liberation of the charge carriers stored at the interfaces and their enhanced migration facilitated by the high frequency of the applied field.^52^ Both the real (*Z*′) and imaginary (*Z*″) parts of the impedance were determined and shown in Fig. S2(a) and (b). Impedance showed the similar frequency dependent behavior like dielectric constant. As in the low frequency range, interfacial polarization is active, and charge accumulation takes place; higher impedance is visible. The gradual declination occurs at high frequency region where charge carriers become incapable of maintaining the polarization with the rapidly oscillating electric field. The Nyquist plot (Fig. S2(c)) exhibited a depressed semi-circle arc with its center below the abscissa axis, confirming non-Debye type relaxation. This supports the idea of charge accumulation at the phase interfaces of Bi_2_O_3_/BiOCl. From the overall dielectric properties of the sample, it can be inferred that the mixed phase interface is highly active in charge accumulation which may have assisted faster charge transfer and reduced the energy barrier for the electron transfer process, thus effectively reducing overpotential and facilitating the overall sensing activity.^53^

**Fig. 6:**
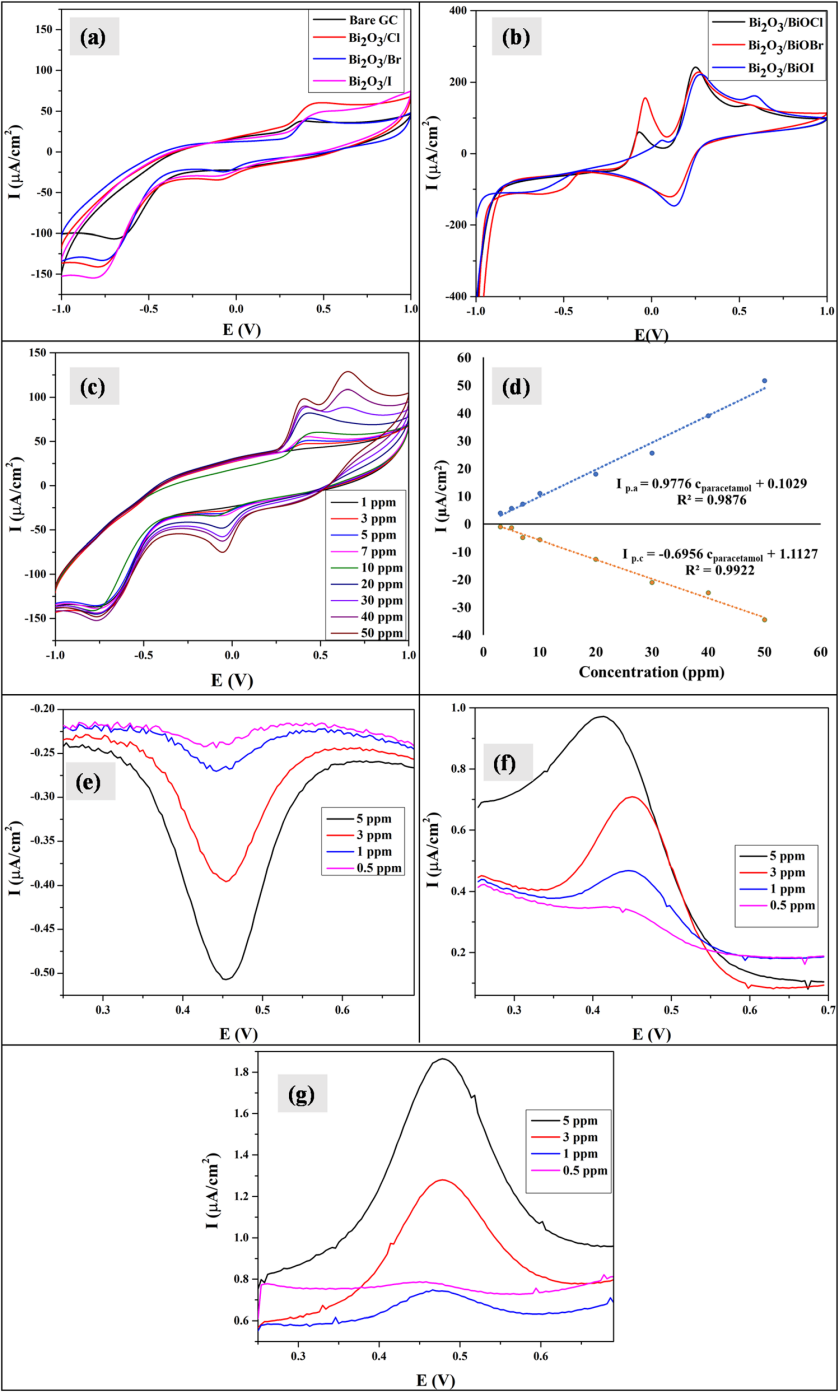
(a) CV of modified and bare GC electrode in 10 ppm paracetamol; (b) CV of the modified electrodes in 0.25 µM solution of [Fe(CN)_6_]^3−/4−^ (c) CV of Bi_2_O_3_/BiOCl modified electrode at different paracetamol concentration; (d) Anodic and cathodic current *vs.* concentration plot; (e) DPV, (f) DNPV and (g) SWV of Bi_2_O_3_/BiOCl modified GC electrode at various concentration levels.

For electrochemical experiments, the modified and unmodified electrodes were first analyzed by cyclic voltammetry (CV) in a 10 ppm paracetamol solution from −1 to 1 V at 25 mV s^−1^ scan rate (Fig. 6(a)). The electrodes showed distinguishable anodic and cathodic peaks when modified with Bi_2_O_3_/BiOCl (*I*_p.a_ = 2.05 × 10^−5^ A cm^−2^, *I*_p.c_ = 8.83 × 10^−6^ A cm^−2^), Bi_2_O_3_/BiOBr (*I*_p.a_ = 1.35 × 10^−5^ A cm^−2^, *I*_p.c_ = 7.29 × 10^−6^ A cm^−2^) and Bi_2_O_3_/BiOI (*I*_p.a_ = 7.52 × 10^−5^ A cm^−2^, *I*_p.c_ = 3.86 × 10^−7^ A cm^−2^), than the bare electrode that only showed an oxidation peak *I*_p.a_ = 6.54 × 10^−6^ A cm^−2^, slightly at a lower potential. Among all, Bi_2_O_3_/Cl showed the highest currents at oxidation and reduction potential.

To measure the surface area, CV was performed in a 0.25 µM solution of [Fe(CN)_6_]^3−/4−^, at 25 mV s^−1^ scan rate from −1 to 1 V potential range (Fig. 6(b)). The “Randles–Sevcik” equation (eqn (5)) was applied in this regard that involves highest anodic or oxidation current (*I*_p.a_), concentration of [Fe(CN)_6_]^3−/4−^ in mole L^−1^ (*C*_0_), active surface area (*A*), quantity of transferred electron in oxidation reaction (*n* = 1), diffusion coefficient (*D* = 7.7 × 10^−6^ cm^2^ s^−1^) and scan rate (*ν*) in V s^−1^.

5 *I*_p.a_ = 2.69 × 10^5^*An*^3/2^*C*_0_*D*^1/2^*ν*^1/2^

Bi_2_O_3_/Cl showed the highest surface area of 3.496 cm^2^, whereas Bi_2_O_3_/Br and Bi_2_O_3_/I showed 2.795 and 2.766 cm^2^ of surface area. Higher peak currents previously found by the Bi_2_O_3_/Cl electrode may have been caused by the larger surface area, as more active space was provided, which facilitated more charge transfer and better interaction.^54^ As Bi_2_O_3_/Cl showed superior characteristics and electrochemical detection compared to other samples, it was chosen for further analysis.

Fig. 6(c) shows the detection of paracetamol at different concentrations (1–50 ppm) by Bi_2_O_3_/Cl using CV under the same conditions of scan rate and potential difference. The highest peaks for oxidation (*I*_p.a_ = 5.16 × 10^−5^ A cm^−2^) and reduction (*I*_p.c_ = −3.45 × 10^−5^ A cm^−2^) were found for 50 ppm at *E*_p.a_ = 0.399 V and *E*_p.c_ = −0.0488 V, respectively. It is evident that, at increasing concentration, both anodic and cathodic peak height increased with a little shift to lower potential and at 1 ppm, there was no visible peak. The phenomenon can be explained by the availability of more paracetamol molecules with growing concentration, which led to a higher rate of oxidation and reduction and eventually showcased higher anodic and cathodic peaks, respectively.^55^ At lower concentrations like 1 ppm, due to the limited availability of electroactive species, compared to the electrolyte and other ions present in solution, a higher proportion of non-faradic to faradaic current may have formed.^56^ For this reason, three different pulse voltammetry techniques were used to neglect the non-faradic part at lower concentrations.^57^ The oxidation and reduction currents were plotted against concentration in Fig. 6(d) which showed equations of *I*_p.a_ (µA) = 0.9776*c*_paracetamol_ (ppm) +0.1029 with *R*^2^ value 0.9876 and *I*_p.c_ (µA) = −0.6956*c*_paracetamol_ (ppm) + 1.1127, *R*^2^ = 0.9922, respectively. This defines the linear relationships between current and concentration in both cases of oxidation and reduction.

For further detection of paracetamol at lower concentration, the oxidation current was evaluated by DPV, DNPV and SWV in 0.5–5 ppm range and visualized in Fig. 6(e)–(g), respectively. For DPV, the voltage range was set at 0.25–0.7 V with increment = 0.004 V, amplitude = 0.025 V and pulse period = 0.7 s. For DNPV, the amplitude was changed to 0.05 V and both the 1st and 2nd pulse time was set at 0.05 s with a pulse period of 0.2 s. For SWV, the amplitude and frequency were set at 0.025 V and 15 Hz, respectively. For all three techniques applied here, the oxidation currents were found to appear with more distinguishable and sharper peaks at these low concentration levels compared to CV. The trend of current increasing with rising concentration resembles that of CV, and more importantly, the 1 ppm as well as the 0.5 ppm solution showed very distinct peaks. This may have become possible due to the pulse applied, which eventually reduced the non-faradic current to a significant level, resulting in increased sensitivity and resolution. The results also demonstrate the compatibility of the synthesized samples with different electrochemical techniques in detecting lower concentrations of paracetamol.

**Fig. 7:**
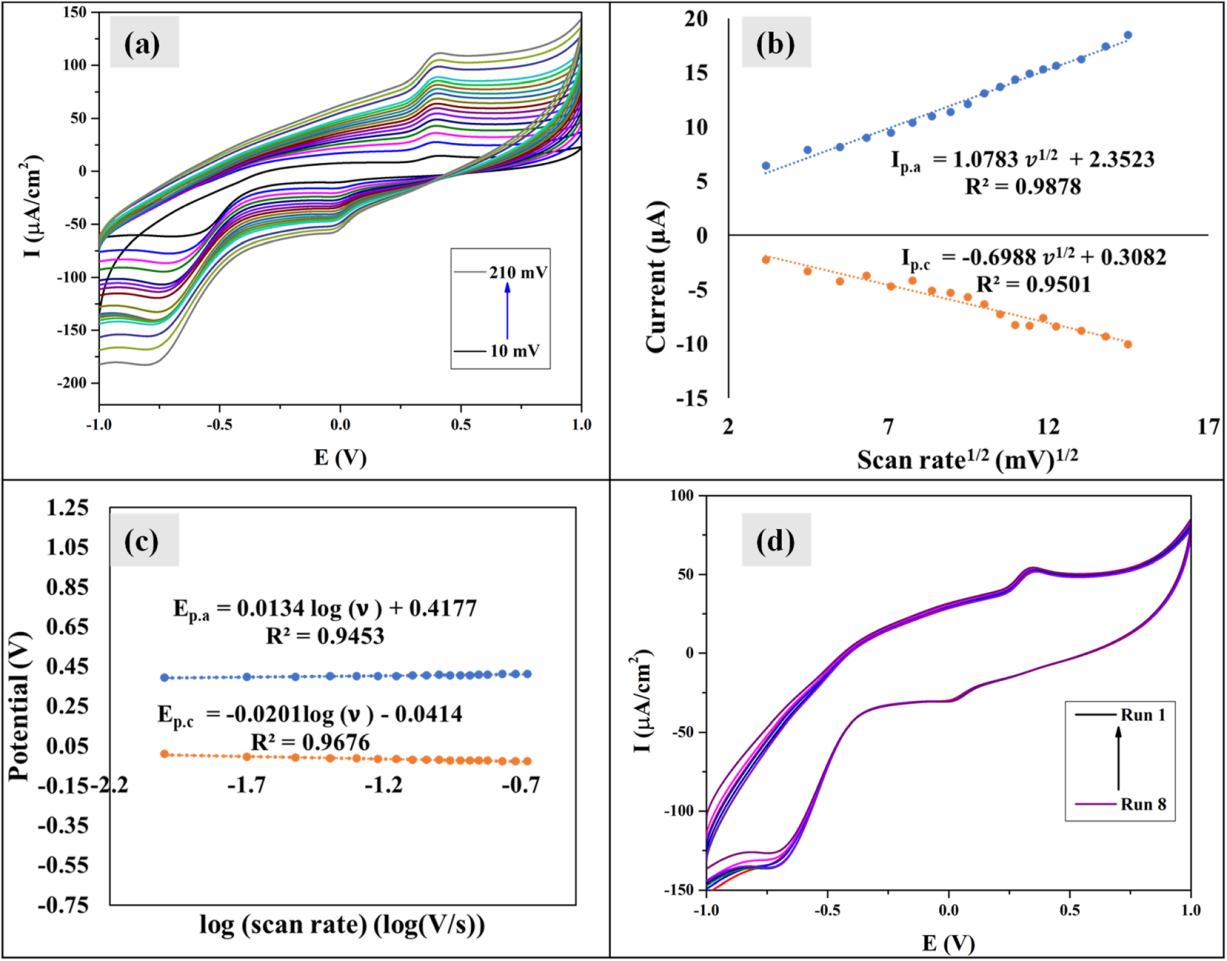
(a) CV of Bi_2_O_3_/BiOCl modified electrode in 10 ppm paracetamol at different scan rates; (b) the relationship of anodic and cathodic current with square root of scan rate; (c) the plots of anodic and cathodic potential *vs.* logarithm of scan rate and (d) repeatability of the process using Bi_2_O_3_/BiOCl electrode.

The scan rate was varied from 10 to 210 mV s^−1^, maintaining the paracetamol concentration at 10 ppm and potential difference at −1 to 1 V (Fig. 7(a)). In this case, the current was found to develop with growing scan rate and a little shift in peak position was noticed. Higher scan rate led to faster potential variation, which eventually resulted in decreasing the size of the diffusion layer and increasing current value.^55^ The plots of oxidation and reduction current *vs.* the square root of scan rate were presented in Fig. 7(b) and 
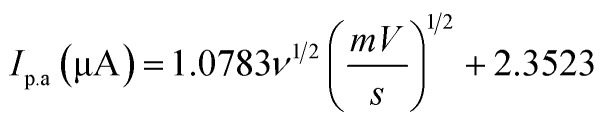
 with *R*^2^ value 0.9878 and 
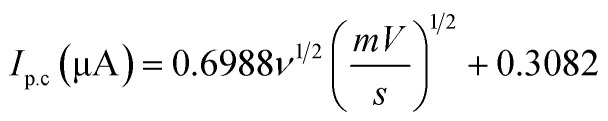
 with *R*^2^ value 0.9501, were found, respectively. The linearity of the plots suggests the electrochemical activity is diffusion-controlled in both cases of oxidation and reduction.^58^Fig. 7(c) shows the plots of oxidation and reduction potential *vs.* logarithm of scan rate from which the equations of *E*_p.a_ = 0.0134 log(*ν*) + 0.4177 (*R*^2^ = 0.9453) and *E*_p.c_ = −0.0201 log(*ν*) − 0.0414 (*R*^2^ = 0.9676) were developed. The linear relationship between potential and logarithm of scan rate indicates the electrochemical process as a quasi-reversible process.^23^ The charge transfer coefficient (*α*) can be calculated from the slopes *k*_a_ = 0.0134 and *k*_c_ = −0.0201 of the *E*_p.a_ and *E*_p.c_*vs.* log(*ν*) plots, respectively. Laviron's theory^59^ states that *k*_a_ and *k*_c_ are equal to 2.3*RT*/(1 − *α*)*nF* and −2.3*RT*/(1 − *α*)*nF*, from which *α* was calculated 0.4 by the following eqn (6):6
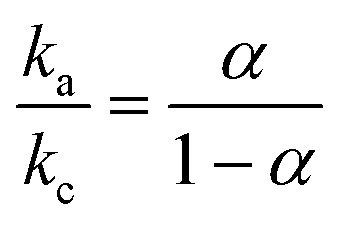


The rate constant of electron transfer, *k*_s_, was also determined by Laviron's theory^59^ provided in eqn (7) as the Δ*E*_p_ were well over 200 mV, indicating a quasi-reversible nature. Here *α* is the charge transfer coefficient, *R* is the molar gas constant, *T* is the temperature, *F* is the Faraday constant, Δ*E*_p_ is peak to peak separation, and *n* is the number of electrons involved in the electrochemical process, which is taken to be 2 for paracetamol according to previous literature.^60^7
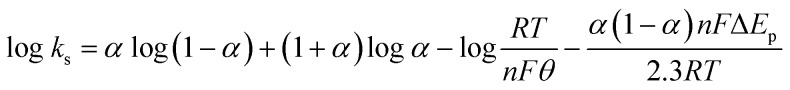


The mean *k*_s_ value was found to be 0.12 cm s^−1^, which denotes a fast electron transfer rate between the Bi_2_O_3_/BiOCl electrode and paracetamol solution.

The repeatability of the electrochemical process was evaluated at a scan rate of 75 mV s^−1^, potential difference −1 to 1 V in a 5 ppm solution and the cycles were repeated 8 times (Fig. 7(d)). No distinguishable change in oxidation or reduction current was found. The standard deviation (SD) was found to be 0.729, from which the LOD and LOQ values were calculated by using 
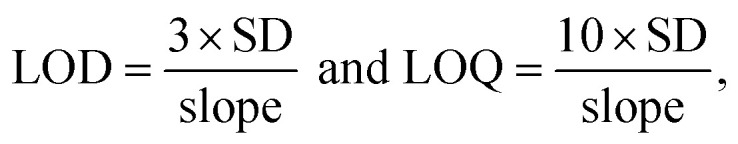
 respectively, where the slope was calculated from the oxidation current *vs.* concentration graph. The LOD and LOQ values were found to be 2.24 ppm and 7.47 ppm, respectively. The sensitivity of the Bi_2_O_3_/BiOCl electrode was also determined from the current *vs.* concentration plot and found to be 0.9776 µA ppm^−1^. The obtained values are compared to other studies and shown in Table 1. The reproducibility of the modified sensor was evaluated using CV (Fig. S3(a)). The assessment was performed using five separately prepared electrodes under identical experimental conditions, yielding an RSD of about 5% which indicates the materials good fabrication consistency. The long-term stability (Fig. S3(b)) of the modified electrode was evaluated by monitoring oxidation peak current during its storage at room temperature. The electrode maintained a stable current response throughout the study, retaining more than 95% of its initial signal after 14 days. No significant decrease in electrochemical performance was detected, indicating good stability of the Bi_2_O_3_/BiOCl sensor.

**Table 1:** Comparison table of paracetamol detection by different modified electrodes

Modified electrode	Electrode type	Electrochemical technique	Linear range	LOD	LOQ	Ref.
Bi_2_O_3_/BiOCl	GC	CV, DPV, DNPV & SWV	1–50 ppm	2.24 ppm	7.47 ppm	This study
			0.5–5 ppm			
Crown ether	CPE	Voltammogram	1–700 µM	28.88 µM	96.28 µM	63
Nafion/TiO_2_ -graphene	GC	CV	1–100 µM	2.1 × 10−7 M	—	66
AuNPs/SDS-LDH	GC	DPV	0.5–400 µM	0.13 µM	—	67
Iron oxide	CPE	DPV	2–150 µM	1.16 µM	—	68
MWCNT/poly (glycine)	GC	CV	5 × 10^−7^ to 1 × 10^−5^ M	5 × 10^−7^ M	1.7 × 10^−6^ M	69
PEDOT/PSSLi	GC	DPV	0.14–400 µM	0.05 µM	—	70
Carbon nanofibers	SPE	DPV	0.09–0.8 mg L^−1^	0.03 mg L^−1^	—	71
gCN-AgPVP	GC	CV	0.2–100 µM	0.079 µM	—	72
Green iron oxide	CPE	LSV	0.8–10 µM	0.287 µM	0.871 µM	73
Boron doped diamond electrode		DPV	20–400 µmol L^−1^	7.1 × 10–6 mol L^−1^	—	74
4-ABA/ERGO	GC	CV	0.1–65 µM	0.01 µM	—	75

**Fig. 8:**
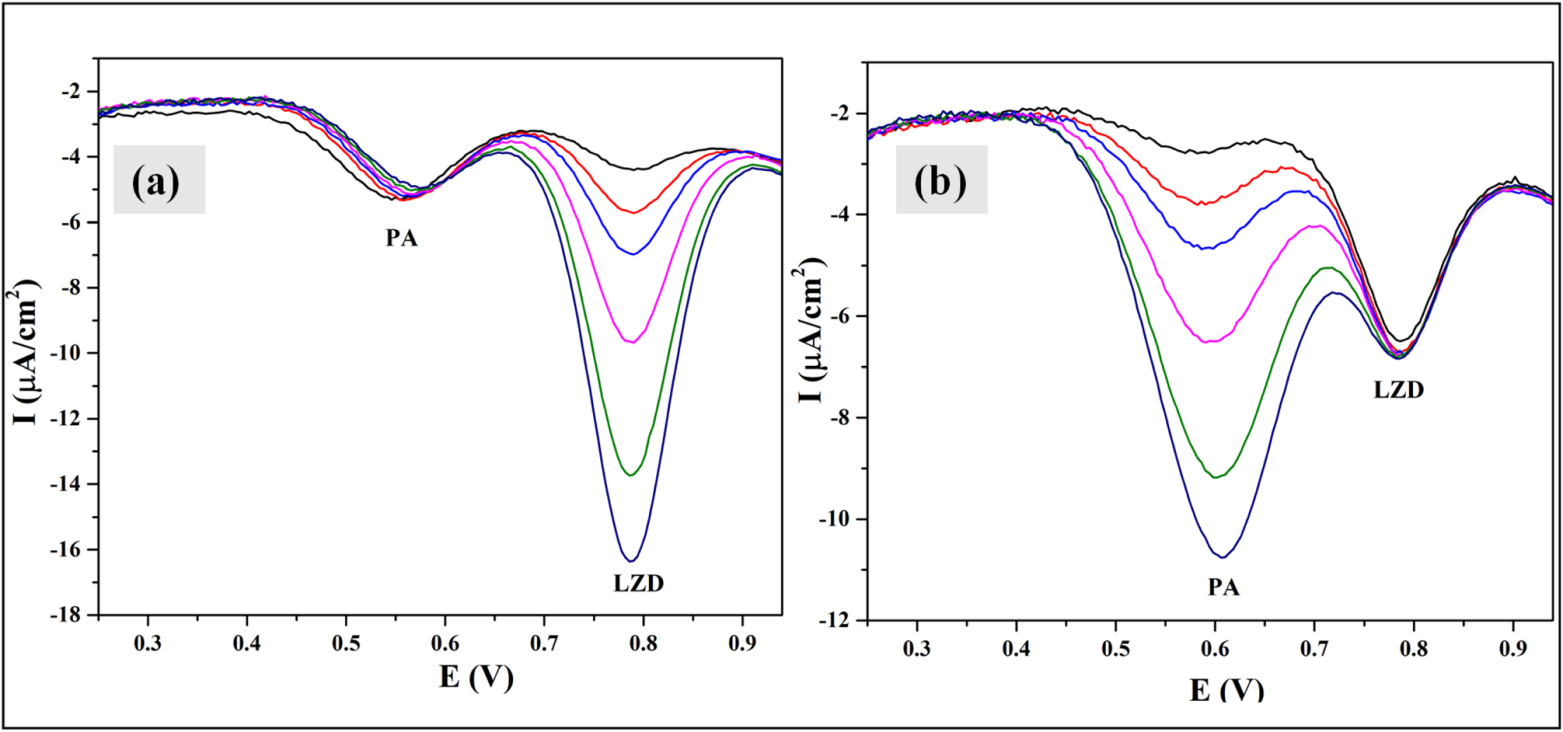
Interference study of Bi_2_O_3_/BiOCl electrode by DPV at (a) concentration variation (1–10 ppm) of LZD under constant concentration of PA at 3 ppm and (b) concentration variation of PA (1–10 ppm) under constant concentration of LZD at 3 ppm.

An interference study was conducted for the simultaneous detection of paracetamol (PA) and linezolid (LZD) using DPV (Fig. 8). To evaluate mutual interference, the concentration of one analyte was systematically varied from 1–10 ppm while keeping the concentration of the other constant at 3 ppm. It was observed that the increasing concentration of PA resulted in a proportional enhancement of its oxidation peak without causing any significant change in the peak position or current response of LZD, and *vice versa*. The distinct and independent oxidation peaks indicate minimal cross-interference between PA and LZD which demonstrates the high selectivity of the modified electrode toward each analyte. These results confirm the feasibility of reliable and simultaneous electrochemical detection of PA in mixed systems.

### 3.1. Proposed mechanism

**Fig. 9:**
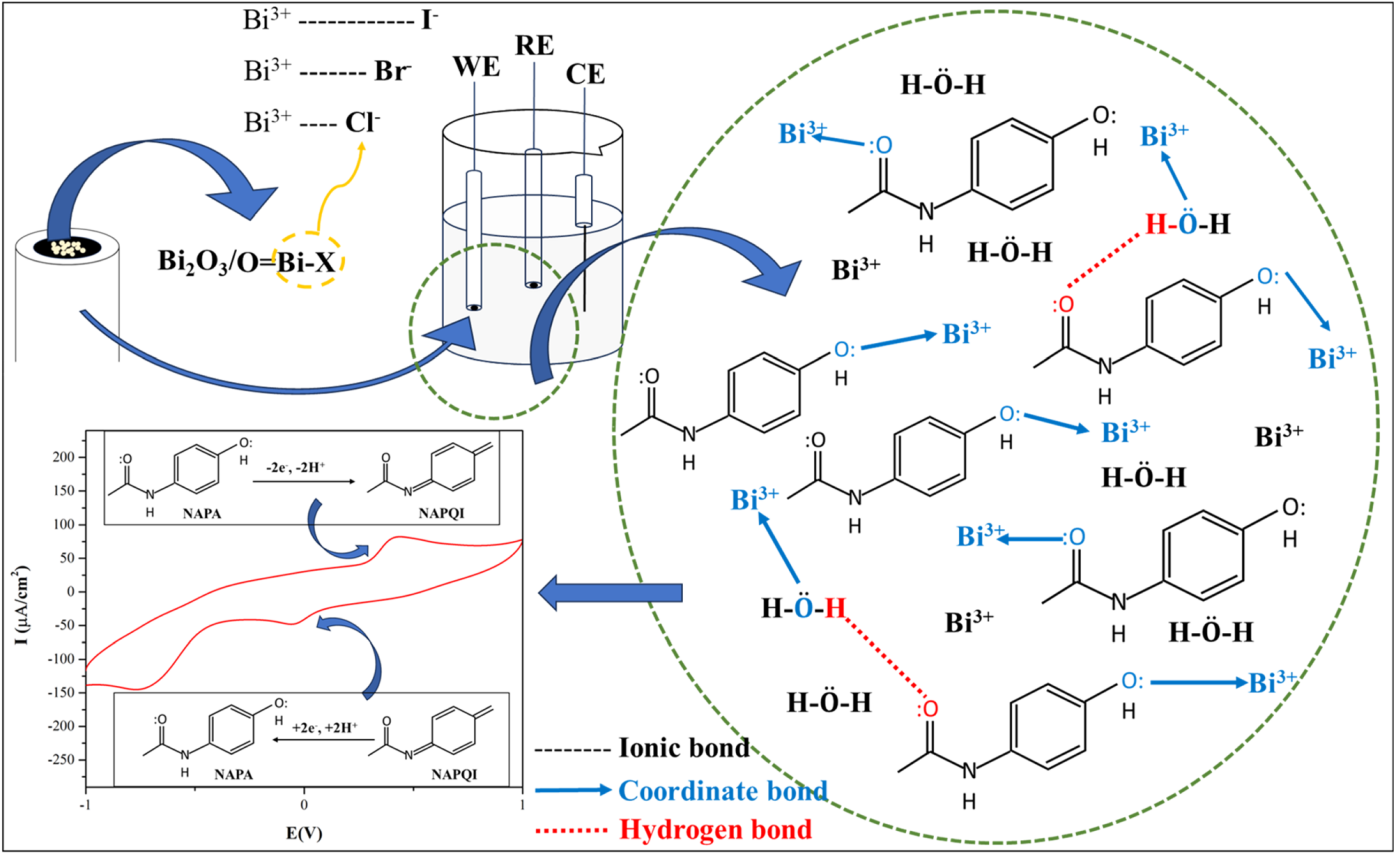
Detection mechanism of Bi_2_O_3_/BiOX modified GC electrode in paracetamol solution using CV.

The variation in the detection level of paracetamol by the synthesized samples may have been caused by the presence of different halide ions. The main bonding between paracetamol and the samples can be considered to be formed by the Bi^3+^ ions (Fig. 9). The interaction can occur directly or indirectly with the paracetamol molecules. In direct interaction, Bi^3+^ may form coordination bonds or complexes with the –OH or –C=O bonds of paracetamol or may indirectly form a stable complex structure with water.^61,62^ The complex may further form a hydrogen bond with the functional groups of paracetamol.^63^ In either case, the bond-forming tendency is influenced by the presence of halides. The bond between Bi^3+^ and halides is primarily ionic but contains some covalent characteristics due to the presence of the Bi 6p orbital, which facilitates orbital overlapping.^64^ As the ions move from Cl^−^ to I^−^, the ionic radius increases and the electronegativity difference with Bi^3+^ decreases, which makes the bonds less ionic and less polar in nature.^65^ Due to these reasons, Bi^3+^ from the Bi–Cl bond might be more electron-deficient and Lewis acidic to accept lone pair electrons from the oxygen of –OH. As the interaction between sensor and paracetamol may be principally due to these chemical interactions, in the case of Bi_2_O_3_/BiOCl, the higher energy band gap may have been compensated by the higher level of interactions. Similarly, Bi_2_O_3_/BiOI may have competed with Bi_2_O_3_/BiOBr by having the lowest energy band gap, which may have caused higher charge transfer corresponding to the results found.

The anodic and cathodic current was produced most likely due to the oxidation of paracetamol or NAPA (*N*-acetyl-*p*-aminophenol) to NAPQI (*N*-acetyl-*p*-benzoquinone-imine) and the reduction of NAPQI back to NAPA, respectively. This mechanism is supported by previous literature,^60^ which also states the probable occurrence of dimerization reaction between NAPQI and the anionic product of NAPA. Due to the formation of the dimer, the amount of reduction may have decreased, which corresponds to the lower amount of cathodic current compared to the anodic current.^23^ At higher concentration (30–50 ppm), the appearance of a second anodic peak at a higher potential can be explained by an additional oxidation step in which previously formed NAPQI may have further oxidized and produced other metabolites like *p*-benzoquinones.

## 4. Conclusion

The current study shows the effective application of mixed-phase Bi_2_O_3_/BiOX samples in paracetamol detection by using different electrochemical methods. Different characterization methods were employed to confirm the presence of both phases in the samples. Under experimental conditions, the sensors demonstrated great sensitivity, with a low detection limit and a dependable quantification range. The modified electrodes showed distinct CV peaks, signifying their possible applicability for quick and precise paracetamol monitoring in environmental or pharmaceutical samples. The findings and mechanism of the process indicate the sensor's potential applicability in sensing different organic compounds and heavy metals as well. The implementation of the Bi_2_O_3_/BiOX modified electrode in real wastewater sample can also be evaluated in future work.

## Author contributions

Raamisa Anjum: methodology, investigation, data curation, formal analysis, writing- original draft, review, and editing. Md. Hasanuzzaman: data curation (TGA &DSC), Muhammad Shahriar Bashar: data curation (SEM & EDX), Juliya Khanam: data curation (Bandgap), Umme Sarmeen Akhtar: data curation (XPS), Sumaya Farhana Kabir: writing-review, supervision. A. M. Sarwaruddin Chowdhury: writing-review, Samina Ahmed: supervision. Md. Sahadat Hossain: conceptualization, resources, methodology, formal analysis, writing-review and editing, supervision.

## Conflicts of interest

There are no conflicts to declare.

## Supplementary Material

RA-016-D6RA01611A-s001

## Data Availability

Data will be made available on request. Supplementary information (SI) is available. See DOI: https://doi.org/10.1039/d6ra01611a.
