# Supplementary material for: Facile synthesis of Bi2O3/BiOX mixed-phase for electrochemical detection of paracetamol
Source: RSC Adv. 2026 Mar 31;16(19):17612–26. doi: 10.1039/d6ra01611a (PMC13037379; doi:10.1039/d6ra01611a)
Supplement: RA-016-D6RA01611A-s001 [file RA-016-D6RA01611A-s001.pdf]

Supplementary Information (SI) for RSC Advances.
This journal is © The Royal Society of Chemistry 2026

Facile Synthesis of Bi_2O_3/BiOX mixed-phase for electrochemical detection of paracetamol

Raamisa Anjum[a,b], Md. Hasanuzzaman[b], Muhammad Shahriar Bashar[c], Juliya Khanam[b], Umme Sarmeen Akhtar[b], A. M. Sarwaruddin Chowdhury[c], Samina Ahmed[b*], Sumaya Farhana Kabir[a*], Md. Sahadat Hossain[b*]

[a]Department of Applied Chemistry and Chemical Engineering, University of Dhaka, Dhaka - 1000, Bangladesh

[b]Institute of Glass & Ceramic Research and Testing, Bangladesh Council of Scientific and Industrial Research (BCSIR), Dhaka 1205, Bangladesh

[c]Institute of Energy Research & Development, Bangladesh Council of Scientific and Industrial Research (BCSIR), Dhaka, Bangladesh

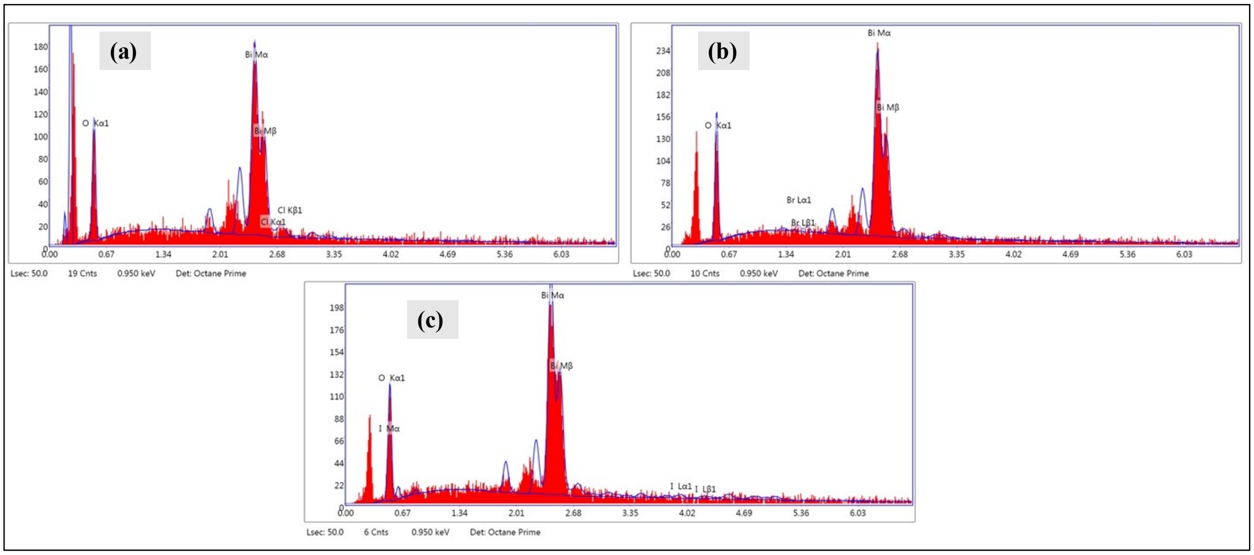

Fig. S1: EDS analysis of (a) Bi_2O_3/BiOCl, (b) Bi_2O_3/BiOBr and (c) Bi_2O_3/BiOI.

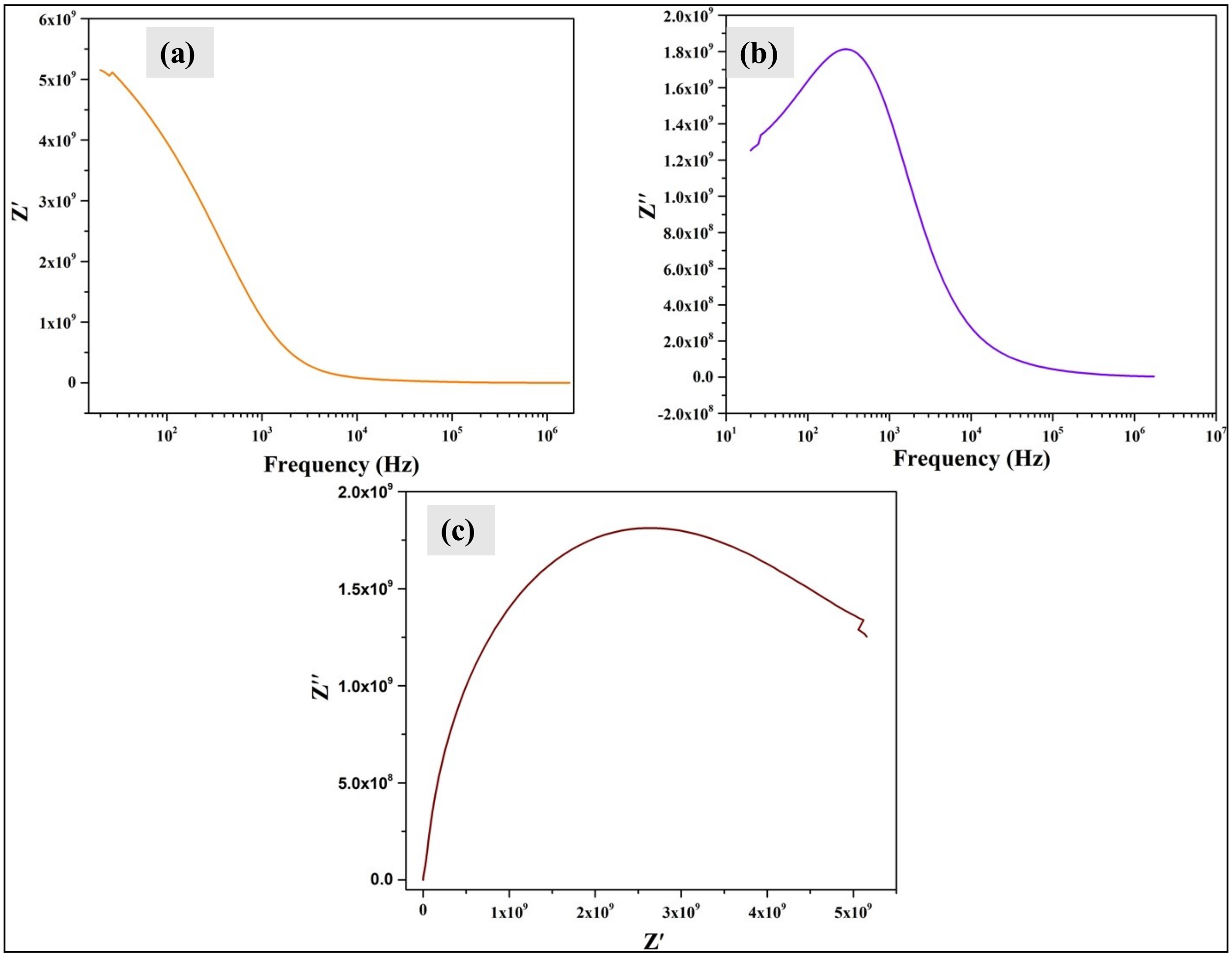

Fig. S2: (a) Real part, Z´ with (b) imaginary part, Z´´ of impedence and (c) Nyquist plot of the Bi_2O_3/BiOCl sample.

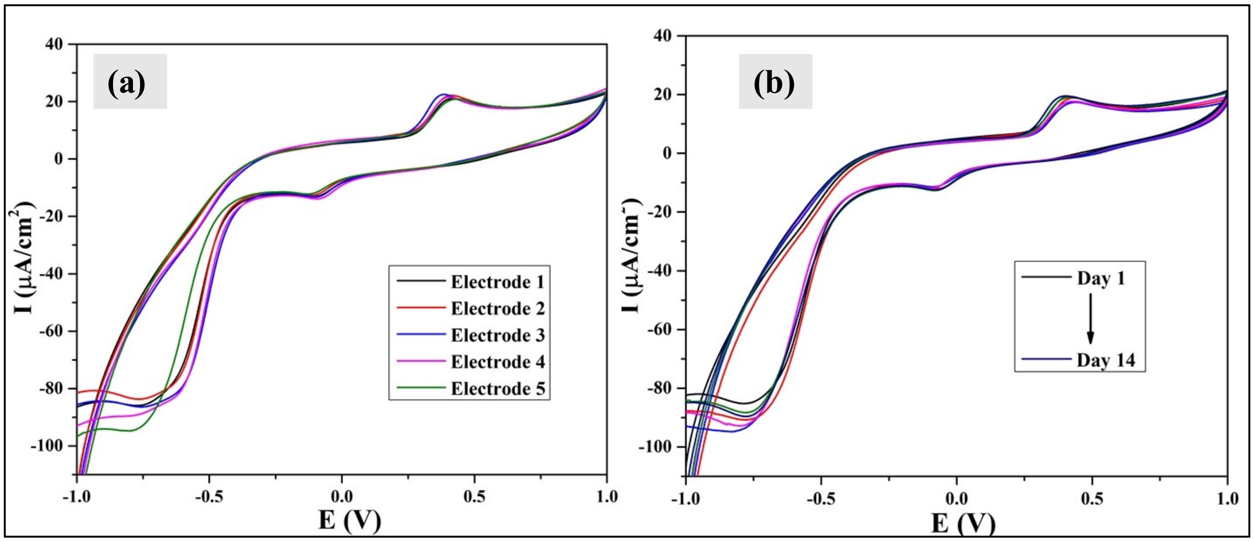

Fig. S3: DPV of Bi_2O_3/BiOCl electrode for (a) reproducibility and (b) stability analysis.
